# Optimizing Printability of Rice Protein‐Based Formulations Using Extrusion‐Based 3D Food Printing

**DOI:** 10.1002/fsn3.4713

**Published:** 2024-12-31

**Authors:** Thuy Trang Nguyen, Safoura Ahmadzadeh, Helmut Schöberl, Ali Ubeyitogullari

**Affiliations:** ^1^ Department of Food Science University of Arkansas Fayetteville Arkansas USA; ^2^ Department of Horticulture and Food Technology Weihenstephan – Triesdorf University of Applied Sciences Freising Germany; ^3^ Department of Agriculture, Food, and Nutrition Weihenstephan – Triesdorf University of Applied Sciences Weidenbach/Triesdorf Germany; ^4^ Department of Biological and Agricultural Engineering University of Arkansas Fayetteville Arkansas USA

**Keywords:** 3D food printing, morphology, protein, rheology, rice, starch

## Abstract

The purpose of this study was to investigate the application of an innovative extrusion‐based 3D food printing (3DFOODP) technique in developing rice protein‐starch (RP‐S) gel‐based products. The effects of 3DFOODP conditions were examined, which included variations in the concentrations of rice protein (RP) and corn starch (S) (15, 17.5, and 20 wt.%), nozzle size (0.8, 1.5, and 2.5 mm), printing temperature (40°C, 60°C, and 80°C), and ingredient flow speed (5.7, 6.3, and 6.9 mL/min). A hollow cylindrical model was chosen as a test object to determine the printability of RP‐S gels. The best 3D printability was achieved using an RP concentration of 17.5% and an S concentration of 15% at 60°C printing temperature with a nozzle size of 1.5 mm, and ingredient flow speed of 6.3 mL/min. With increasing the RP concentration, a rise in apparent viscosity, loss, and storage moduli was observed. The recovery test showed the gels' rapid and reversible response. The freeze‐dried 3D‐printed RP‐S gels showed a porous granular structure, depending on the printing temperature. No chemical interactions between the RP and S were observed as analyzed by FTIR. Overall, RP, in combination with S, provides a new opportunity for the 3DFOODP and their utilization by the alternative protein industry.

## Graphical abstract

This study investigated the use of rice proteins for 3D food printing applications. The 3D printing parameters (i.e., concentration, nozzle size, printing temperature, and ingredient flow speed) were optimized for the best shape fidelity. The developed 3D printing approach could increase the utilization of rice proteins in the alternative protein industry.

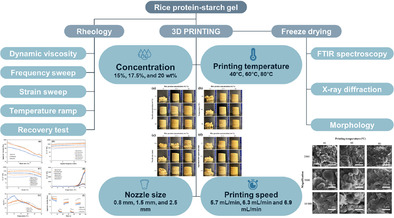

## 1 Introduction

3DFOODP involves layer‐by‐layer deposition of materials to create 3D objects, and it has the potential to revolutionize the food industry (Le‐Bail, Maniglia, and Le‐Bail 2020; Pereira, Barroso, and Gil 2021). Companies like Aleph Farms (K. Handral et al. 2022) and Meatech (Steenhuis, Fang, and Ulusemre 2022; Tibrewal, Dandekar, and Jain 2023) have embraced 3DFOODP for meat production innovations. Simultaneously, it has facilitated the growth of plant‐based alternatives, as exemplified by Redefine Meat (Tibrewal, Dandekar, and Jain 2023) and Novameat (Peshave and Hodlur 2022).

A critical factor for 3DFOODP is the printability of food materials, which depends on certain properties, such as melting, glass transition temperature, gelatinization/gelation, and shear‐thinning behavior (Liu et al. 2017; Tan et al. 2018; Sehgal, Singh, and Sharma 2022). Previous 3DFOODP research explored a range of materials, including mashed potatoes (Liu et al. 2018), cookie dough formulations (Jagadiswaran et al. 2021), lemon juice gel and potato starch (Yang et al. 2018), fish surimi gel and NaCl (Wang et al. 2018). Generally, the printability of these materials have been enhanced with additives, such as gums (Azam et al. 2018; Strother, Moss, and McSweeney 2020), starch (Chen et al. 2019; Dong et al. 2019), pectin (Vancauwenberghe et al. 2019; Agarwal, Costantini, and Maiti 2021), gelatin (Strother, Moss, and McSweeney 2020; Du et al. 2021), nanocellulose (Ee and Yau Li 2021), alginate (Liu et al. 2020), carrageenan (Kim et al. 2022), and other substances (Tan et al. 2018; Piyush, Kumar and Kumar 2020).

Recently, there has been growing interest in utilizing plant proteins in food formulations for 3D printing. However, plant proteins from a single source generally lack certain essential amino acids, leading to concerns about a lower nutritional value compared to animal proteins. This concern primarily arises from their deficient levels of essential amino acids, particularly lysine, methionine, histidine, and isoleucine (Dimina et al. 2022). Consequently, legumes and cereal proteins are used together in food formulations, as they are known to complement each other in terms of their amino acid profiles. While the 3D printability of plant proteins from plant protein sources like soy (Ainis et al. 2023), zein (Rowat, Legge, and Moresoli 2021), pea (Kim, Kim, and Park 2021), cowpea (Mune Mune, Minka, and Mbome 2014) oat and faba bean (Lille et al. 2018), common bean (Shi et al. 2022), and mushroom (Keerthana et al. 2020) have been previously reported, information regarding the 3D printability of cereal proteins remains limited.

Rice stands as a staple food in many parts of the world, playing a predominant role as a foundational cereal grain within Asian nations and the United States (Al‐Doury, Hettiarachchy, and Horax 2018). As the leading rice producer in the United States, Arkansas accounts for 50.1% of the nation's total rice production and commands 49.8% of the total rice acreage planted in 2022 (Hardke, Sha, and Bateman 2023). RP, accounting for around 7%–9% of their total weight, can be obtained from the endosperm of the kernel or the bran. Both sources are acknowledged for their hypoallergenic, nutritional, and health benefits. In addition, RP is gluten‐free (Amagliani et al. 2017), positioning it as a competitive protein substitute in the food ingredient industry.

RP emerges as a premier cereal‐based protein due to its lysine content, which is second only to oats (Al‐Doury, Hettiarachchy, and Horax 2018; Jayaprakash et al. 2022). Rice contains a total of 18 amino acids; among them, 8 are essential, including isoleucine, leucine, lysine, methionine, phenylalanine, threonine, tryptophan, and valine. Considering the high demand for plant‐based foods, RP has received great attention due to its attributes, including its bland flavor, hypoallergenic properties, gluten‐free nature, preferred amino acid profile, and lysine content (Shin, Gang, and Song 2010).

There have been few studies that report on the use of rice protein blended with other plant proteins and hydrocolloids as printing inks to create printed products. Barrios‐Rodríguez et al. (2024) studied the effect of 3D printing parameters such as printing speed, nozzle diameter, and layer height on protein food development utilizing rice protein and xanthan gum. Their findings revealed that layer height and nozzle diameter both have a substantial impact on printing precision, shape stability, surface quality, resolution, and layer arrangement. Lower printability ratings were associated with lower speed (20 mm/s) and a larger nozzle diameter (2.2 mm), which resulted in greater deformities in the final protein cylinders. In another study, Qiu et al. (2023) created 3D‐printed meat analogs utilizing a high‐protein printing ink made from soy protein isolate, wheat gluten, and rice protein. They also evaluated the edible inks' rheological characteristics and printing performance. The authors reported that raising the rice protein fraction decreased the apparent viscosity and storage modulus of the printing inks, which enhanced the printability.

To the best of our knowledge, there is no existing study on the 3D printability of starch‐rice protein mixtures using an extrusion‐based 3D food printer. Moreover, the printability of rice protein has not been extensively investigated. Consequently, the main objective of this study was to investigate and optimize the 3D printability of RP using extrusion‐based 3DFOODP. Specific objectives were to (i) optimize the 3D printing parameters (i.e., RP/S concentration, printing temperature, and printing speed), and (ii) characterize the RP‐based formulations (i.e., rheology) and 3D‐printed objects (i.e., texture, morphology, and chemical structure). The use of rice proteins as 3D printing materials provides a new opportunity to expand their current use by increasing their utilization in the alternative protein industry.

## 2 Materials and Methods

### 2.1 Materials

Rice protein was kindly provided by Axiom Foods (CA, USA). High amylose corn starch (72% amylose content) was supplied by Ingredion (IL, USA).

### 2.2 Preparation of Rice Protein‐Starch Gel Formulations

RP‐S gels were prepared in nine different formulations (Table 1). First, dispersions comprising 15%, 17.5%, and 20% (w/w) RP were prepared. Next, the samples were homogenized using a VWR ULTRA‐TURRAX homogenizer for 10 min at 13,500 rpm (Wu et al. 2022). The pH level of the solution was subsequently raised to 8 by adding 1 M sodium hydroxide. Afterward, the solutions were placed in an ice bath and ultrasonicated for 20 min (Cole Parmer, IL, USA). The ultrasonicator was run with the following settings: a duty cycle of 70%, pulse widths of 5.0 and 1.0, and a temperature of 20°C. After adding S at 15%, 17.5%, and 20% (w/w), the pH was readjusted to 8 to ensure the solubility of proteins. The mixture was then left in a water bath at 90°C for 30 min. This heating process contributed to protein denaturation, exposing further functional groups that may affect the protein's interchain interactions, in addition to the S gelatinization. After incubation, the resulting gel was carefully loaded onto the printing cartridges (50 mL) for 3D printing.

**TABLE 1:** 3D printing samples with different rice protein (RP) and starch (S) concentrations.

Rice protein concentration (wt.%)	Starch concentration (wt.%)	Sample code
15	15	RP15:S15
17.5	15	RP17.5:S15
20	15	RP20:S15
15	17.5	RP15:S17.5
17.5	17.5	RP17.5:S17.5
20	17.5	RP20:S17.5
15	20	RP15:S20
17.5	20	RP17.5:S20
20	20	RP20:S20

### 2.3 3D Food Printing of Rice Protein‐Starch Gels

**FIGURE 1:**
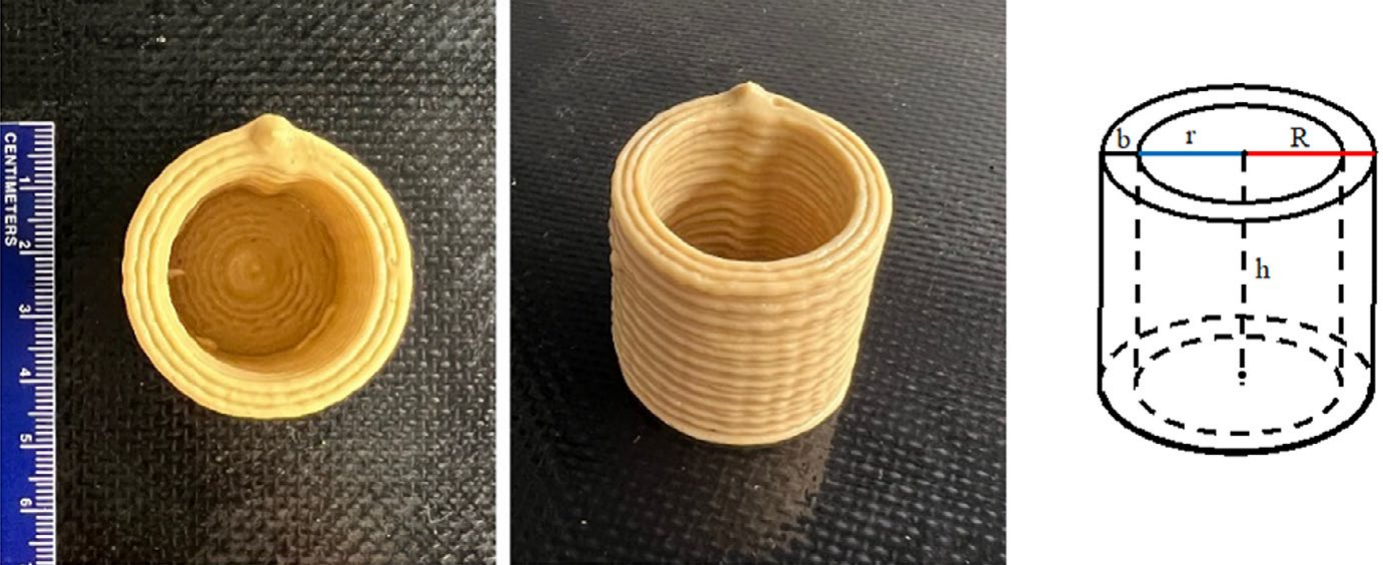
3D‐printed cylinder consisting of RP and S and hollow cylindric model with height (*h*) = 3.5 cm, internal radius (*r*) = 1.5 cm, external radius (*R*) = 2.0 cm, and thickness (*b*) = 0.5 cm.

RP‐S gels were printed onto 19 × 28 cm silicone‐coated Mylar plastic sheets (Richard Mistler Inc., PA, USA) using a 3D food printer (Foodini, Natural Machines, Spain). For 3D printing, a cylindrical model with dimensions of height (*h*) = 3.5 cm, internal radius (*r*) = 1.5 cm, external radius (*R*) = 2 cm, and thickness (*b*) = 0.5 cm was selected (Figure 1). The volume of the model was 19.2 cm^3^. The following printing parameters were set on the 3D printer: print speed of 3200 mm/min, and layer height of 1.4 mm. The printing parameters (i.e., RP concentration (15%, 17.5%, and 20% [w/w]), S concentration (15%, 17.5%, and 20% [w/w]), printing temperature (40°C, 60°C, and 80°C), nozzle size (0.8, 1.5, and 2.5 mm), ingredient flow speed (5.7, 6.3, and 6.9 mL/min) were optimized consequently).

The RP‐S gels obtained by 3D printing were transferred to a −80°C ultra‐low temperature freezer for 24 h. The gel was then dried at −108°C for 48 h using a freeze dryer (VirTis Benchtop SLC, PA, USA). After drying, the freeze‐dried gels were immediately transferred to airtight containers until future use (Ahmadzadeh and Ubeyitogullari 2023).

### 2.4 Rheological Properties

The rheological properties of the RP‐S gels were evaluated using a controlled‐stress rheometer (AR 2000 Rheometer; TA Instruments, DE, USA) equipped with a 40 mm parallel plate geometry, as described by Ahmadzadeh and Ubeyitogullari (2023). The measurements were performed immediately after heating the samples at 90°C for 30 min in a water bath. A thin layer of silicone oil was applied around the perimeter to prevent the sample from drying during the analysis. The viscosity, oscillation tests, and shear recoverability tests were conducted at a constant temperature of 60°C. The temperature ramp tests were performed from 25°C to 90°C, where 2°C/min, 0.01%, and 10 rad/s adjustments were set as the temperature rate, strain, and frequency, respectively. The dynamic moduli as a function of frequency were recorded and used to conduct the angular frequency sweeps. A rotational recovery test was used to examine the shear recovery. The test was divided into five phases: each lasting 180, 120, 180, 120, and 180 s with shear rates of 1, 100, 1, 100, and 1 1/s, respectively (Ji et al. 2022).

### 2.5 Morphology of the Freeze‐Dried Samples

Post‐print photographs of both top and front views were taken with a digital camera (i.e., iPhone 13 Pro, CA, USA). The dimensional properties of the prints, namely the height and radius of the cylindrical structure, were carefully determined by using a ruler as a reference.

Freeze‐dried RP‐S gels, pure S, and RP were analyzed using scanning electron microscopy (SEM) imaging. This was accomplished by using a FEI Nova Nanolab 200 Dual‐Beam system equipped with a 30 kV SEM FEG column and a 30 kV FIB column following the method described by Ahmadzadeh and Ubeyitogullari (2022). To obtain a suitable sample, thin cross‐sections were skillfully cut from the 3D‐printed gels and coated with a thin layer of gold using an EMITECH SC7620 sputter coater (MA, USA). SEM images were then taken in the 800–10,000 magnification range while maintaining an accelerating voltage of 15.0 kV and a current of 10 mA.

### 2.6 X‐Ray Diffraction

The X‐ray diffraction (XRD) patterns of freeze‐dried RP‐S gels 3D‐printed at different temperatures (40°C, 60°C, and 80°C) were investigated utilizing a PW3040 X'Pert MRD High‐Resolution XRD instrument (manufactured by Philips, Almelo, Netherlands). The powdered RP‐S gels were scanned between 5° and 45°. During the XRD measurements, the instrument was configured with a voltage of 45 kV and an electric current of 40 mA, while employing a scan step size of 0.02°. An identical experimental protocol was employed to investigate the properties of pure RP and S for comparison purposes. Furthermore, the crystallinity was calculated using OriginPro 2023 software (OriginLab Corporation, MA, USA). The crystallinity index was determined by dividing the area under the crystalline peaks by the total area under the peaks (i.e., crystalline and amorphous peaks).

### 2.7 Fourier Transform Infrared (FTIR) Spectroscopy

The freeze‐dried RP‐S gels 3D‐printed at various printing temperatures (40°C, 60°C, and 80°C) were ground before the FTIR analysis. The structural features of the RP‐S gels were investigated using an IRAffinity‐1S Fourier transform infrared spectroscopy (FTIR) unit (SHIMADZU Corp., Kyoto, Japan) equipped with a Quest attenuated total reflectance (ATR) accessory (Specac Company, Orpington, UK). The FTIR spectrum was acquired in the range of 4000–500 cm^−1^ at a resolution of 4 cm^−1^ with 64 scans.

### 2.8 Statistical Analysis

The statistical analysis was conducted using Minitab 16.1.1 software (Minitab Inc., PA, USA). The statistical differences among the treatments were determined using analysis of variance (ANOVA) and Tukey's multiple comparison test at a significance level of *p* < 0.05. The data are presented as mean ± standard deviation. All the experiments were performed in triplicates.

## 3 Results and Discussion

### 3.1 3DFOODP Of RP‐S Gels

The ideal 3D printing material possesses a well‐defined network structure, high gel strength, proper viscosity, and the capacity to be printed below the extrusion pressure. Moreover, it should seamlessly fuse with previously printed layers to maintain the intended printing shape. During extrusion, there is high shear stress, causing a decrease in viscosity for smoother flow, which is why 3DFOODP requires materials with shear‐thinning behavior (Lanaro et al. 2017; Lenie et al. 2024). The viscosity should be low enough to go through the nozzle but high enough to be cohesive and maintain the form of the object without deforming previous layers (Liu et al. 2018).

#### 3.1.1 Effect of Concentration

**FIGURE 2:**
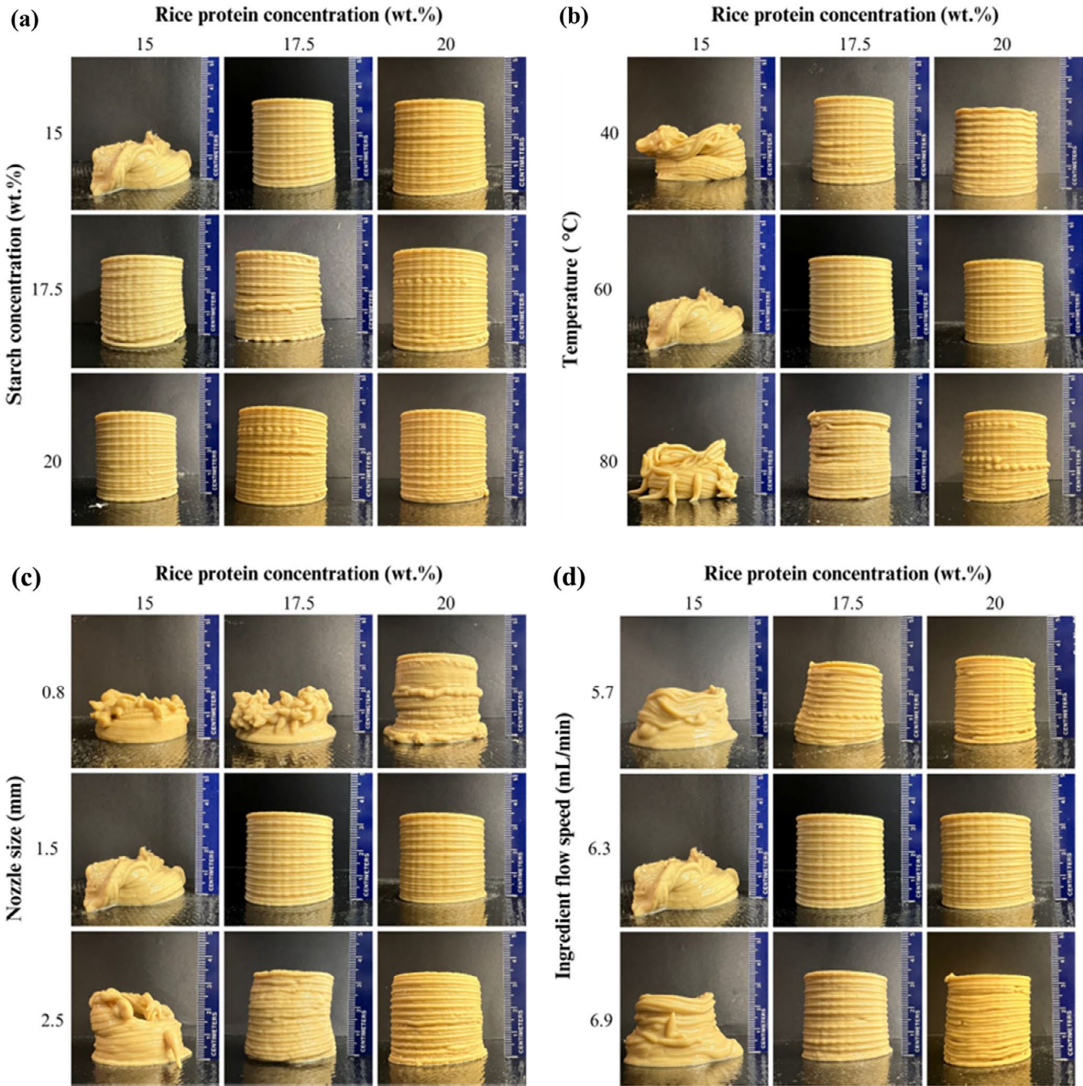
Pictures of (a) RP‐S gels 3D printed with various concentrations (printing temperature: 60°C, nozzle size: 1.5 mm, and flow speed: 6.3 mL/min), (b) RP‐S gels 3D printed with constant S concentration of 15 wt.% at various RP concentrations and printing temperatures (nozzle size: 1.5 mm, and flow speed: 6.3 mL/min), (c) RP‐S gels 3D printed with constant S concentration of 15 wt.% at various RP concentrations and nozzle sizes (printing temperature: 60°C, and flow speed: 6.3 mL/min), and (d) RP‐S gels 3D printed with constant S concentration of 15 wt.% at various RP concentrations and ingredient flow speeds (printing temperature: 60°C, and nozzle size: 1.5 mm). RP, Rice protein; S, starch.

The stability of the shape was influenced by the printing process as well as the material used. Figure 2a presents pictures of 3D prints using different concentrations of RP and S. These pictures were compared with a focus on external flaws to assess the printing results. A successful print is one with a stable shape, characterized by uniform and aligned layers. The concentration of materials is key to obtaining a stable print with minimized flaws. Setting the concentration too high could clog the nozzle, while setting it too low could result in poor control of the deposition of the material. To determine the optimal concentration combination, the printability values were compared (Figure 2a, Table 2).

**TABLE 2:** The volumes and heights of the digital 3D model and the 3D‐printed samples.

3D model		Volume (cm^3^)											
		19.2											
		S (%)			Temperature (°C)			Nozzle size (mm)			Flow speed (mL/min)		
		15	17.5	20	40	60	80	0.8	1.5	2.5	5.7	6.3	6.9
RP (%)	15	NA	19.1 ± 1.5^bA^	19.1 ± 1.2^bA^	NA	NA	NA	NA	NA	NA	NA	NA	NA
	17.5	19.4 ± 0.6^aB^	20.8 ± 1.0^bAB^	23 ± 1.4^aA^	18.1 ± 1.4^bA^	19.4 ± 0.6^aA^	18.8 ± 1.2^bA^	NA	19.4 ± 0.6^aA^	21.3 ± 1.6^aA^	12 ± 3^bB^	19.4 ± 0.6^aA^	19.7 ± 1.0^aA^
	20	19.1 ± 0.8^aB^	23.7 ± 1.3^aA^	21.1 ± 1.5^abB^	20.1 ± 0.4^aA^	18.7 ± 1.2^aA^	20.2 ± 1.6^aA^	12.6 ± 1.4^B^	19 ± 1.2^aA^	19.6 ± 0.3^aA^	18.3 ± 1.2^aA^	18.3 ± 0.4^aA^	18 ± 0.8^aA^

3D model		Height (cm)											
		3.5											
		Starch (%)			Temperature (°C)			Nozzle size (mm)			Flow speed (mL/min)		
		15	17.5	20	40	60	80	0.8	1.5	2.5	5.7	6.3	6.9
RP (%)	15	NA	3.5 ± 0.2^bA^	3.4 ± 0.2^bA^	NA	NA	NA	NA	NA	NA	NA	NA	NA
	17.5	3.5 ± 0.2^aA^	3.3 ± 0.5^abA^	3.8 ± 0.2^aA^	3.3 ± 0.2^aA^	3.5 ± 0.2^aA^	3.5 ± 0.6^aA^	NA	3.5 ± 0.2^aA^	3.6 ± 0.3^aA^	3.3 ± 0.4^aA^	3.5 ± 0.2^aA^	3.5 ± 0.1^bA^
	20	3.6 ± 0.2^aB^	3.9 ± 0.1^aA^	3.6 ± 0.1^abB^	3.4 ± 0.6^aA^	3.4 ± 0.4^aA^	3.4 ± 0.3^aA^	3.5 ± 0.7^A^	3.7 ± 0.3^aA^	3.7 ± 0.1^aA^	3.5 ± 0.2^aA^	3.7 ± 0.1^aA^	3.7 ± 0.2^aA^

*Note:* Means with the same capital letter within the same row for the same parameter are not significantly different (*p* < 0.05), and means with the same lowercase letter within the same column for the same parameter are not significantly different (*p* < 0.05).

Abbreviations: NA, not applicable; RP, rice protein; S, starch.

All concentration combinations were printable except for the one with the least concentration of 15% RP and S, RP15:S15. This can be observed in the collapsed shape, where the ability to bear the height was not satisfactory. The best printing result was achieved with an RP concentration of 17.5% and an S concentration of 15% (RP17.5:S15). Specifically, the volume and height of the 3D‐printed samples were also determined and compared to the 3D model (Table 2). This measurement, combined with a visual assessment of shape accuracy and resolution, assisted in the evaluation of printing accuracy. Variances in volume and height from the 3D model, whether higher or lower, indicated a lower build quality. RP17.5:S15 with the least defects in cylindrical geometry closely aligned with the volume and height of the 3D model (19.4 cm^3^, and 3.5 cm for the 3D print compared to 19.2 cm^3^, and 3.5 cm for the 3D model) had the best printing quality. As a result, RP17.5:S15 was selected based on its high printability value and its high RP content (17.5%) and low S content (15%). As the focus of this study was on the printability of RP, we maintained the S concentration at 15% and investigated the other 3D printing parameters (i.e., temperature, nozzle size, and ingredient flow speed) at varying protein concentrations. Similar results were obtained by Carvajal‐Mena et al. (2023), where the effects of salmon protein isolate and S concentrations on the printability of starch‐protein gels were investigated.

#### 3.1.2 Effect of Temperature

Figure 2b shows the effect of the temperature on the 3D printing quality of gels prepared using 15%–20% RP and 15% S. When 15% RP was used, the 3D‐printed objects were not able to hold their shapes at all the printing temperatures investigated. At the printing temperature of 40°C, the printing layers had some irregularities, which could be due to the strong gel formed upon cooling to 40°C. On the other hand, the layers were not able to maintain their shape fidelity during printing at 80°C, which could be due to the decreased viscosity, preventing the gels' ability to hold the printed layers. Printing temperature of 60°C provided the best shape fidelity in both RP concentration of 17.5% and 20% (Figure 2b). However, Table 2 shows that at 60°C, 17.5% RP had a volume of 19.4 cm^3^ with an average height of 3.5 cm. This was more consistent with the 3D model, demonstrating improved printing quality when compared to 20% RP.

According to Lian et al. (2024), temperature affects the morphology of alkali‐induced RP gels. They reported that the heat treatment at 80°C and the time spent at this temperature had a visible effect on the gel. The gels demonstrated significantly lower viscosity by increasing the time of treatment at 80°C, which is consistent with our findings. Lian et al. (2024) suggested that heat treatment increased integration between glutelin particles in the gel network, which could be directly related to the gel's viscosity reduction and the change in microstructure. Therefore, the impact of temperature on the microstructure, crystallinity, and chemical interactions was investigated (see Sections 3.3, 3.5).

#### 3.1.3 Effect of Nozzle Size

The nozzle size has a direct impact on the precision and surface texture of the 3D print (Yang et al. 2018). The effects of three different nozzle sizes (0.8, 1.5, and 2.5 mm) were evaluated using 15% S along with varying concentrations of RP (15%–20%) (Figure 2c). The 3D printability of the samples with a nozzle size of 0.8 mm was poor in all three RP concentrations (i.e., 15%, 17.5%, and 20%) due to nozzle clogging, preventing continuous printing to completion. The small diameter (0.8 mm) caused an increased shear‐thinning behavior and a decrease in viscosity, which influenced the flowability and deformation (Oliveira et al. 2020). Even though the first couple of layers during printing with a 0.8 mm nozzle seemed uniform and integrated well (i.e., individual layers cannot be differentiated), they could not support the shape along with other issues described above (e.g., nozzle clogging). Nevertheless, when the nozzle size was increased to 1.5 mm, the printability of the samples significantly improved, especially when 17.5% and 20% RP were used. Still, the RP15:S15 sample was not able to hold its shape due to the low concentrations of the materials. However, with a larger nozzle size of 2.5 mm, all three 3D prints with RP concentrations of 15%, 17.5%, and 20% had poor printability. This resulted from the gradual reduction in the distance between the deposited gel and the moving nozzle's tip as the product accumulated. As a result, the originally intended design could not be formed accurately on the platform. Furthermore, when comparing printing accuracy measurements, 3D printing of 17.5% RP with a 1.5 mm nozzle size resulted in volume and height measurements that closely matched those of the 3D model (Table 2), suggesting the optimum concentration.

#### 3.1.4 Effect of Ingredient Flow Speed

The ingredient flow speed explains the rate of deposition of the printing material on the platform (Severini et al. 2018; Jagadiswaran et al. 2021). Changing this parameter did not enhance the 3D printability of RP15:S15 (Figure 2d). The height of the shape remained low at 5.7 mL/min printing speed (Table 2). The shape of RP17.5:S15 with 5.7 mL/min speed was fully printed but tilted (Figure 2d). When the flow speed was increased to 6.3 mL/min, the printabilities of RP17.5:S15 and RP20:S15 were significantly improved (*p* < 0.05). The volume and height measurements (Table 2) confirmed that 17.5% RP provided the best printing quality at 6.3 mL/min flow speed. Nevertheless, increasing the flow speed further to 6.9 mL/min negatively affected the print quality with overlapping layers (i.e., over‐extrusion and increased deposition), causing poor shape fidelity.

### 3.2 Rheological Properties

#### 3.2.1 Viscosity

**FIGURE 3:**
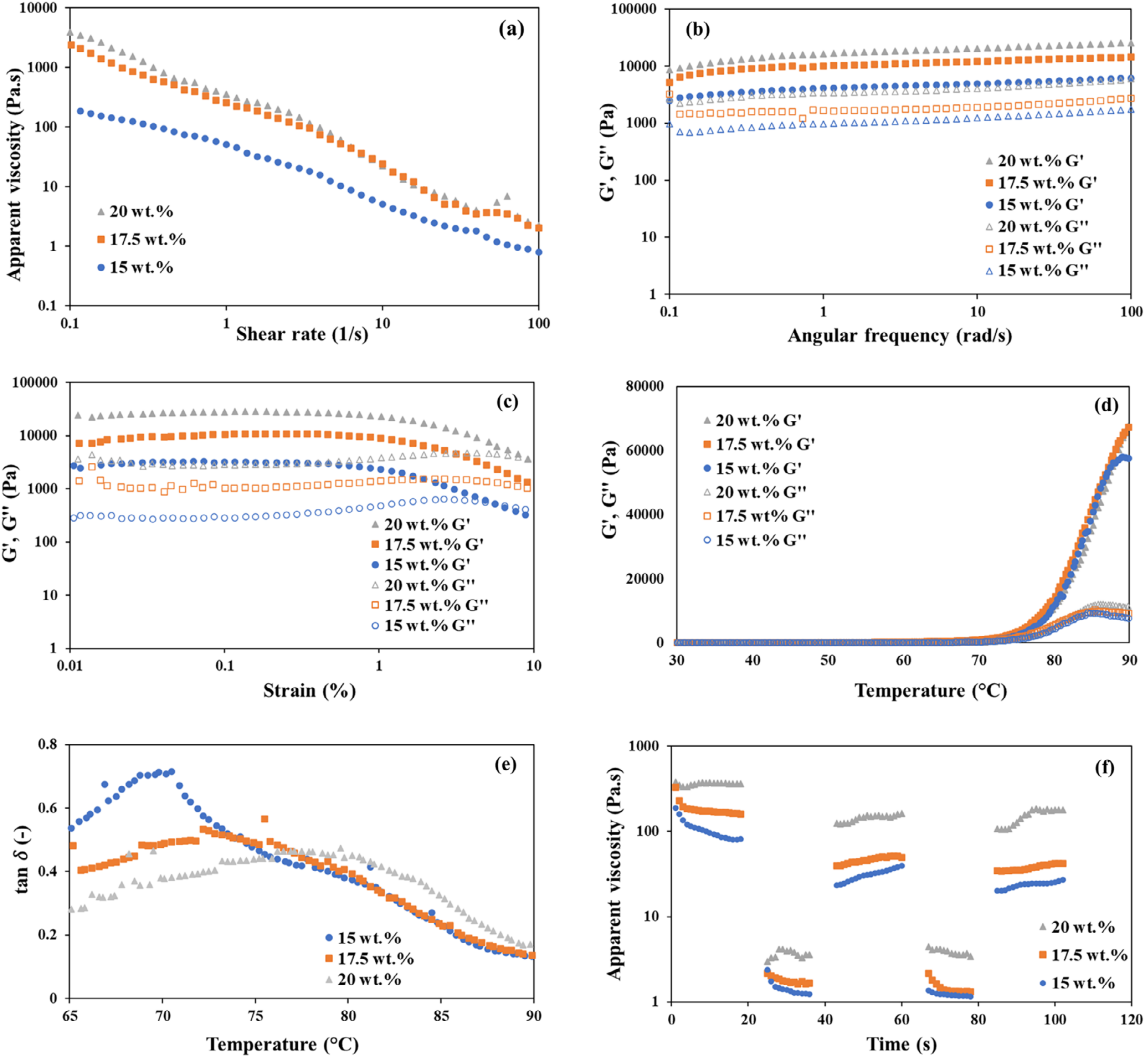
(a) Apparent viscosity as a function of shear rate, (b) storage (*G*′), and loss (*G*″) moduli as a function of angular frequency, (c) strain sweep curves, (d) temperature ramp cues, (e) loss tangent as a function of temperature, and (f) recovery tests of RP‐S gels at a S concentration of 15 wt.% and RP concentrations of 15, 17.5, and 20 wt.%.

Figure 3a shows the dynamic viscosity curves of RP‐S gels, illustrating their viscosity change as a function of shear rate. An increase in shear stress led to a decrease in viscosity for all samples, indicating a shear‐thinning behavior. The shear‐thinning behavior is important for extrusion‐based 3D printing because the ink is subjected to higher shear stresses during extrusion (i.e., at the nozzle tip), resulting in reduced viscosity, which allows for smooth and controlled extrusion. Among the tested RP concentrations, RP17.5:S15 and RP20:S15 provided better printability than RP15:S15, which can be related to their higher apparent viscosities (Figure 3a). At higher protein concentrations (17.5% and 20% vs. 15%), a denser network structure was obtained due to the lower amount of free water (Eliasson 1986). For example, in a previous study, incorporating higher amounts of fish proteins into S gel increased protein‐starch matrix viscosity. This was linked to increased protein matrix density and the growth of elastic starch granules (Wang et al. 2018).

#### 3.2.2 Frequency, Strain, and Temperature Sweep Tests

Figure 3b presents the frequency sweep curve, showing storage and loss modulus as a function of angular frequency (rad/s). The *G*′ defines the elastic behavior, while *G*″ represents the viscous response when changing frequency. *G*′ was higher than *G*″ in all samples (Figure 3b), indicating the elastic behavior of the gels was dominant, which can benefit the shape fidelity after 3D printing. With higher frequency, the viscoelastic properties did not change drastically, indicating a weak frequency dependence. However, with higher RP concentration, a rise in *G*′ and *G*″ was observed.

The low *G*′ of RP15:S15 resulted in poor shape fidelity of the 3D‐printed objects due to the low mechanical strength of the material. In contrast, the higher *G*′ of RP17.5:S15 and RP20:S15 provided proper mechanical strength to support the 3D‐printed layers (Figure 2a).

Figure 3c represents the strain sweep curves of RP15:S15, RP17.5:S15, and RP20:S15, where *G*′ was higher than *G*″ for all the samples. In the range of 0.01 to around 1% strain, both *G*′ and *G*″ remained relatively constant, indicating that the material exhibited a stable viscoelastic behavior in this low strain range. While at around 1% strain, it can be observed that *G*′ decreased while *G*″ increased slightly, so the material exhibited more viscous behavior, and its elastic deformation was reduced. The network structure was disrupted, and the material began to deform more plastically while having weaker elastic properties. The network structure was further disrupted because the intermolecular bindings were weakened, and the density of entanglements within the material was reduced (Liu et al. 2021).

The mechanical properties of the gel and the transition from elastic to viscous behavior under strain are crucial because the change can have implications for 3D printing, particularly when it comes to the ink's ability to maintain a desired shape. Therefore, if the material becomes too viscous and loses its elasticity during printing, the structures could be distorted or poorly defined. Furthermore, the flowability and extrudability are also affected by the disruption of the network within the material. That can have an impact on the flow through the nozzle and adhesiveness to previously printed layers, influencing the overall printing accuracy.

The viscoelastic properties of RP15:S15, RP17.5:S15, and RP20:S15 as a function of temperature are depicted in Figure 3d. At temperatures below 75°C, there was no significant change in the rheological properties as the gelatinization temperature of S was not reached yet. The S granules remained intact and did not start to form a gel network. As the temperature increased, decreases in *G*′ and *G*″ were observed. A 3D gel network was formed by leached‐out amylose and strong interactions among the swollen S particles (Hsu, Lu, and Huang 2000). Figure 3e reveals a loss tangent behavior in which *G*′ exceeded *G*′′ in all cases, demonstrating that by raising the temperature above 80°C, a continuous network was formed, resulting in a dramatic increase in *G*′ and a significant decrease in tan *δ* (Ahmadzadeh and Ubeyitogullari 2023). This indicates an elastic solid behavior characterized by a gel structure that is suitable for 3D printing. Furthermore, as the concentrations of RP increased, the gels' loss tangent values decreased, indicating a more solid behavior and improved ability to retain their shape over time. However, this also led to the formation of small broken lines, which can be observed in the shapes with RP and S of 20 wt.% (Figure 2a) (Wang et al. 2018).

Amylose is released when S granules swell internally, causing changes in rheological properties. As these granules swell and rupture, releasing amylopectin fragments into the dispersed S matrix, they serve as structural fillers. This phenomenon increases *G*′ and *G*′′ values while decreasing the loss tangent value (Bakar et al. 2009).

#### 3.2.3 Recovery Test

The recovery test is important because it replicates the shearing conditions while printing. To understand how these individual forces affect viscosity changes, shear recovery tests were conducted (Figure 3f). The viscosity of all inks decreased at a high shear rate of 100 1/s but quickly recovered when the shear rate was reduced to 1 1/s. The gels' structure recovery varied significantly, as evidenced by the different percentages of final viscosity recoverability. Reduced protein content (from 20% to 17.5%–15%) in the gels resulted in a significant reduction in recoverability, which ranged from 46.6% in the RP20:S15 gel to 23.0% in RP17.5:S15, and 24.0% in the RP15:S15 gel. In addition, the material with the highest RP concentration exhibited the highest viscosity, with a consistent order from highest to lowest RP concentration, which matches the observations obtained from the viscosity curve in Figure 3a.

This reversible viscoelastic behavior enables the ink to extrude easily at high shearing rates and subsequently regain sufficient mechanical strength to support the deposition of subsequent layers (Liu et al. 2019). The phenomenon of the reversible nature of S networks can be explained by the polymer conformational change theory, saying that flexible S macromolecular chains orient along the flow direction under external stress and reduce the conformational entropy of the polymer system. When the external stress is removed, the entropy is partially restored, leading to rapid recovery at low shear (Shaw 2011).

These results indicated that gels containing 15% S and 17.5%/20% RP were suited for 3D printing due to their good structural recoverability and improved support capability upon deposition onto the printing platform.

### 3.3 Morphology of the Freeze‐Dried Samples

**FIGURE 4:**
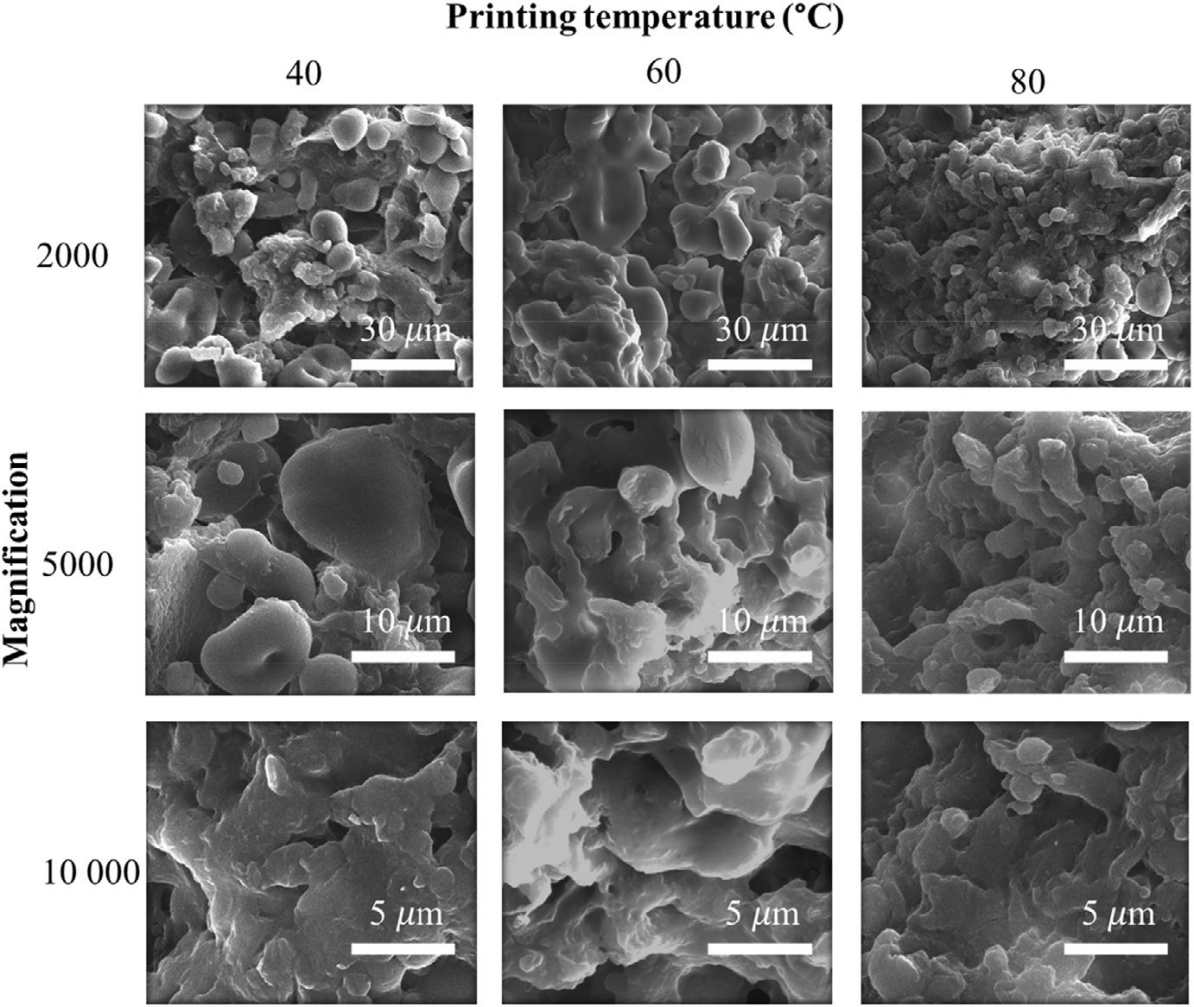
SEM images of freeze‐dried 3D‐printed rice protein‐starch (RP‐S) gels consisting of 15 wt.% S and 17.5 wt.% RP concentration using a nozzle size of 1.5 mm at various printing temperatures.

The effect of temperature on the microstructure was investigated further as the temperature was expected to impact the gelation/gelatinization of the polymers (Figure 4). The microstructures of the gels formed at 60°C and 80°C substantially differed from those formed at 40°C.

The SEM images revealed the presence of spherical aggregates in all samples (Figure 4). Notably, the cross‐section of the gel network appeared substantially rougher following printing at 40°C compared to printing at 80°C, and smaller particles were visible at higher temperatures. By increasing the printing temperature, the printed object's cross‐section structure became more compact and smoother. With 80°C printing temperature, the protein particles' intermolecular binding was relatively tight, and a layer of S matrix appeared to form around the connected protein particles. These findings suggest that the heat treatment facilitated the fusion of glutelin particles inside the gel network, potentially contributing to the gel's lower viscosity (Figure 2b) (Ji et al. 2022). On the other hand, at 80°C, the S formed a stable 3D network and entrapped the protein particles because of the gradual gelatinization of S (Chen et al. 2022).

### 3.4 X‐Ray Diffraction

**FIGURE 5:**
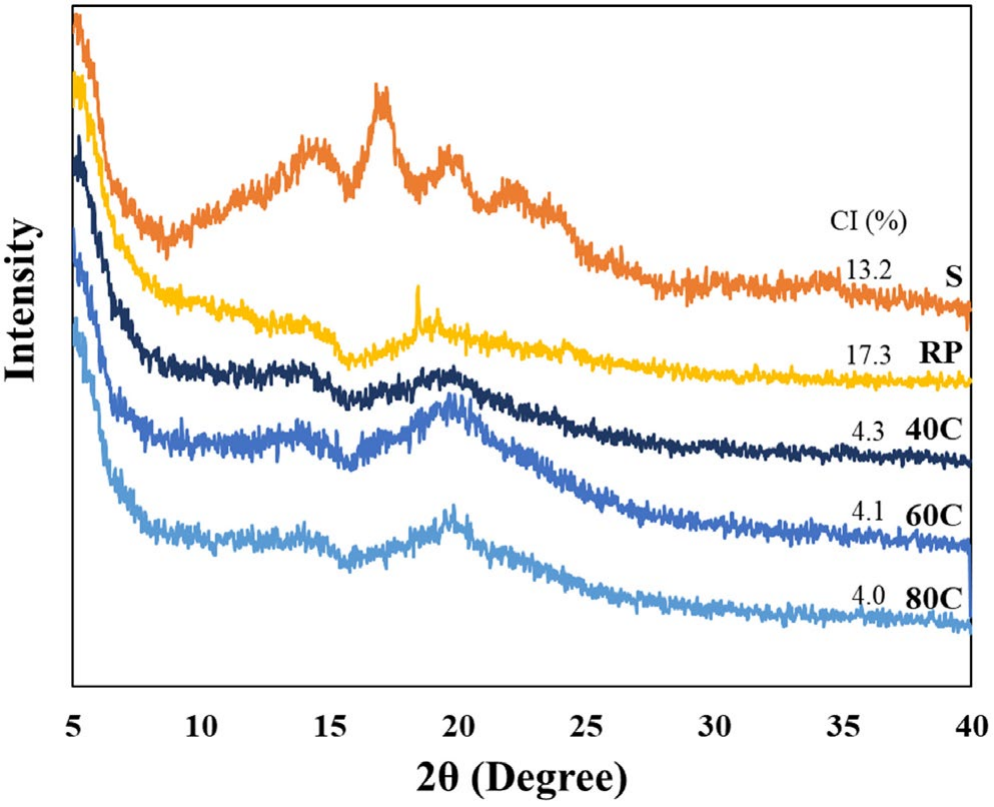
XRD patterns of (S) starch, (RP) rice protein, and freeze‐dried 3D‐printed RP17.5:S15 gels produced using printing temperatures of (40°C) 40°C, (60°C) 60°C, and (80°C) 80°C. CI, Crystallinity index.

The crystalline structure of RP, S, and the RP‐S gels printed at 40°C, 60°C, and 80°C were determined using XRD analysis. As depicted in Figure 5, the native S exhibited a typical V‐type XRD pattern with prominent peaks at 2*θ* = 14.5°, 17.1°, 19.8°, 22.2° and 23.9°, with a crystallinity of 13.2% (Ahmadzadeh and Ubeyitogullari 2023). Meanwhile, RP showed diffraction peaks at 2*θ* = 14.8°, 18.5°, 19.3° and 24.4° (Figure 5), with a crystallinity of 17.3%. The freeze‐dried gels of RP17.5:S15 exhibited a diffraction peak at 2*θ* close to 17° as a result of gelatinization and retrogradation (Chen et al. 2014). This could be attributed in part to the complex formation between amylose and RP (Wu et al. 2023).

The high peak intensity of pure S was attributed to its intact crystalline regions (i.e., crystallinity of 13.2%). In contrast, the RP‐S gel underwent gelatinization, where the S experienced a disruption in its crystallinity. The RP17.5:S15 gels exhibited crystallinities of 4.0%, 4.1%, and 4.3% when printing temperatures of 80°C, 60°C, and 40°C, respectively, were employed. It can be speculated that the gelatinization/gelation was improved with the increase in the printing temperature, explaining the reduction in crystallinity. Similar observations were reported by Dong and Cui (2021).

### 3.5 Fourier Transform Infrared (FTIR) Spectroscopy

**FIGURE 6:**
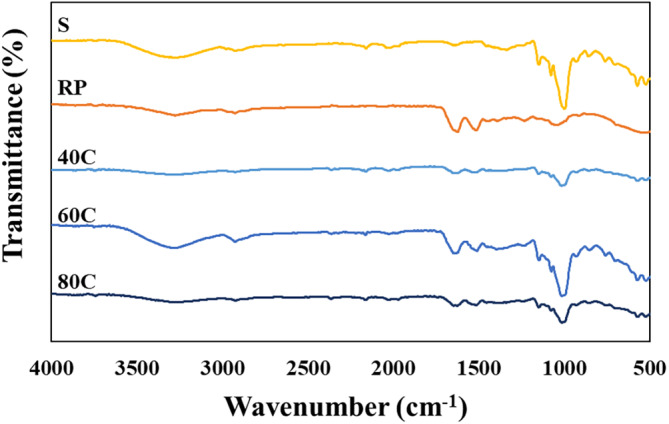
ATR‐FTIR spectra of (S) starch, (RP) rice protein, and freeze‐dried 3D‐printed RP17.5:S15 gels produced using printing temperatures of (40°C) 40°C, (60°C) 60°C, and (80°C) 80°C.

FTIR spectroscopy provided insights into the molecular interactions between S and RP, ultimately confirming the formation of the RP‐S gels (Figure 6). The FTIR spectrum of S showed characteristic bands of S at 3200–3400 cm^−1^ (–OH stretching), 2900–3000 cm^−1^ (C–H stretching), 1100–1150 cm^−1^ (C–O, C–C and C–O–H stretching), and 1100–900 cm^−1^ (C–O–H bending) (Warren, Gidley, and Flanagan 2016). On the other hand, the FTIR spectrum of RP revealed characteristic peaks at 1624 cm^−1^, 1514 cm^−1^, and 1230 cm^−1^, which were associated with amide I (C=O Stretching), amide II (N–H bending), and amide III (C–N stretching), respectively (Dong and Cui 2021).

In the 1200–800 cm^−1^ range, the gel spectrum showed three distinct peaks at 1047, 1022, and 995 cm^−1^. These peaks establish both ordered and disordered configurations of S. Starch's ordered structure which was represented by the absorption peak at 1047 cm^−1^, whereas its disordered composition was represented by the absorption peak at 1022 cm^−1^. Furthermore, the absorbance at 995 cm^−1^ indicated the hydrogen bond structure of S molecules (Zhang et al. 2019). The degree of order in the S was represented by the absorbance peaks ratio of 1047/1022 cm^−1^ (Sevenou et al. 2002). As the printing temperature was reduced, the 1047/1022 cm^−1^ ratio increased, indicating that the interactions among the S molecules increased, resulting in an increase in the degree of order. This was consistent with the results of the XRD analysis (Figure 5).

Upon comparing the gel's spectrum with that of native S or RP, no new bands were identified in the RP‐S gels compared to pure RP or S, which can be associated with the absence of covalent interactions within the gel structure (Liu et al. 2023). The results agreed with the studies of potato starch/soy protein and rice starch/casein complexes (Dong and Cui 2021; Liu et al. 2023).

## 4 Conclusions

The RP‐S gels were successfully prepared at different concentrations, and their 3D printability was optimized. The optimal printing conditions were achieved with a formulation of 17.5% RP and 15% S, a printing temperature of 60°C, a nozzle size of 1.5 mm, and an ingredient flow speed of 6.3 mL/min. These gels exhibited shear‐thinning behavior, where viscosity, as well as loss and storage moduli, increased as the RP concentration increased. The recovery test demonstrated the rapid and reversible viscosity response of the RP‐S gels. The printing temperature notably impacted the microstructure of the 3D‐printed freeze‐dried RP‐S gels. The crystallinity of RP‐S gels was reduced compared to that of pure S and RP. No chemical interactions between RP and S were observed. In conclusion, the combination of RP and S opens new opportunities in 3DFOODP, expanding their current applications and enhancing their potential use in the alternative protein industry.

## Author Contributions

**Thuy Trang Nguyen:** methodology, investigation, formal analysis, writing – original draft. **Safoura Ahmadzadeh:** methodology, formal analysis, investigation, writing – original draft. **Helmut Schöberl:** supervision, writing – review and editing. **Ali Ubeyitogullari:** conceptualization, methodology, investigation, resources, supervision, writing – review and editing, project administration, funding acquisition.

## Conflicts of Interest

The authors declare no conflicts of interest.

## Data Availability

The authors have nothing to report.
